# Solid-State Synthesis and Photocatalytic Activity of Polyterthiophene Derivatives/TiO_2_ Nanocomposites

**DOI:** 10.3390/ma7053786

**Published:** 2014-05-14

**Authors:** Ruxangul Jamal, Yakupjan Osman, Adalet Rahman, Ahmat Ali, Yu Zhang, Tursun Abdiryim

**Affiliations:** 1 Key Laboratory of Petroleum and Gas Fine Chemicals, Educational Ministry of China, College of Chemistry and Chemical Engineering, Xinjiang University, Urumqi 830046, Xinjiang, China; E-Mails: jruxangul@sina.com (R.J.); jakobosm@gmail.com (Y.O.); Adaletr@sohu.com (A.R.); ahmatjanchem@126.com (A.A.); zhyurainy@163.com (Y.Z.); 2 Key Laboratory of Functional Polymers, Xinjiang University, Urumqi 830046, Xinjiang, China

**Keywords:** polyterthiophene derivative, nano-TiO_2_, solid-state method, photocatalyst

## Abstract

Poly(3,4-propylenedioxy-2,2′:5′,2″-terthiophene)/TiO_2_ and poly(3,4-(2,2-dimethylenepropylenedioxy)-2,2′:5′,2″-terthiophene)/TiO_2_ nanocomposites were synthesized by a simple solid-state method. Additionally, the poly(3,4-propylenedioxy thiophene)/TiO_2_ and poly(3,4-2,2-dimethylenepropylenedioxythiophene)/TiO_2_ nanocomposites were synthesized in a similar manner for comparison. The structure and morphology were characterized by Fourier transform infrared (FTIR), ultraviolet-visible (UV-Vis) absorption spectroscopy, X-ray diffraction (XRD) and transmission electron microscopy (TEM). The photocatalytic activities of the nanocomposites were examined through the degradation processes of a methylene blue (MB) solution under UV light and sunlight irradiation. The results of FTIR and UV-Vis spectra showed that the composites were successfully synthesized by solid-state method and the poly(3,4-propylenedioxy-2,2′:5′,2″-terthiophene)/TiO_2_ and poly(3,4-(2,2-dimethylenepropylenedioxy)-2,2′:5′,2″-terthiophene)/TiO_2_ nanocomposite had a higher oxidation degree and conjugation length than others. The results also indicated that the TiO_2_ had no effect on the crystallinity of composites, but was well embedded in the polymer matrix. Additionally, the highest degradation efficiency of 90.5% occurred in the case of the poly(3,4-propylenedioxy-2,2′:5′,2″-terthiophene)/TiO_2_ nanocomposite.

## 1. Introduction

The use of semiconducting materials in environmental decontamination as photocatalysts has attracted a great deal of attention in recent years [1]. When semiconducting materials are exposed to light with energy larger than their band gaps, electron-hole pairs can be created, which can initiate photocatalytic reactions [2]. Most photocatalytic systems are based on TiO_2_, which is chemically stable and has a long life-time of electron-hole pairs generated by optical excitation. However, the band gap of TiO_2_ is ~3.2 eV, which is in the ultraviolet regime, and therefore, visible light, which comprises most of the solar light, cannot be absorbed by TiO_2_ [3]. To overcome this problem, some strategies have been investigated, including noble metal deposition, the doping of metal or nonmetal ions, mixing with another metal oxide, surface photosensitization with dye and preparing composites with a conducting polymer [4–6]. Conducting polymers have attracted considerable attention, because of their interesting semiconducting, electronic and optical properties [7]. Among conducting polymers, polyaniline and polythiophene are widely used for the fabricating of conducting polymer/TiO_2_ hybrid materials [8,9]. Polythiophene and its derivatives show many advantages in combining with TiO_2_, and the large internal interface area in the polythiophene/TiO_2_ composite enables an efficient separation of charge, which is very important for photovoltaic application [10,11]. At the present time, numbers of reports have been published on the preparation of polythiophene and its derivatives/TiO_2_ composites, including original *in situ* photopolymerization [12], electrochemical polymerization [13] and the chemical solution method [14]. Although polythiophene has become the focus of considerable interest, polythiophene suffers from the occurrence of undesired α,β- and β,β’-couplings during polymerization, which deteriorate their electronic and optical properties [15,16]. As derivatives of polythiophene, polyterthiophene-type conjugated polymers have rapidly gained considerable attention, due to the preexistence of α,α’-linkages in their monomers, which makes the whole polyterthiophene-type chain grow regularly and leads to very interesting electronic, electrochromic and optical properties [17–19]. Additionally, they can be prepared by electrochemical and chemical methods. Recently, we have demonstrated a novel room-temperature solid-state oxidative method for the polymerization of ethylenedioxy-substituted terthiophene, and we have found that solid-state polymerization was an effective method for the polymerization of the terthiophene-type monomer [20].

In this paper, we selected 3,4-propylenedioxy-2,2′:5′,2″-terthiophene and 3,4-(2,2-dimethylenepropylenedioxy)-2,2′:5′,2″-terthiophene as monomers for the preparation of the polyterthiophene derivatives/TiO_2_ nanocomposites. As these monomers have α,α’-linkages, it can be deduced that the resulting polymers may grow regularly and will have a high absorption coefficient and a wide absorption wavelength in the visible part of the spectrum, which will allow the TiO_2_ photocatalyst to harvest incident light efficiently in composite photocatalyst. Furthermore, these monomers are in a solid state, and they can be polymerized by the solid-state method. In the present work, with the aim of the above consideration, the room-temperature solid-state polymerization method was applied for the oxidative polymerization of the above terthiophene monomers, 3,4-propylenedioxy-2,2′:5′,2″-terthiophene (TPT) and 3,4-(2,2-dimethylenepropylenedioxy)-2,2’:5’,2″-terthiophene (TMPT), for the preparation of the poly(3,4-propylenedioxy-2,2′:5′,2″-terthiophene)/TiO_2_ (poly(TPT)/TiO_2_) and poly(3,4-(2,2-dimethylenepropylene-dioxy)-2,2′:5′,2″-terthiophene)/TiO_2_ (poly(TMPT)/TiO_2_) nanocomposites. Additionally, the poly(3,4-propy-lenedioxythiophene)/TiO_2_ (PProDOT/TiO_2_) and poly(3,4-2,2-dimethylene-propylenedioxythiophene)/TiO_2_ (PProDOT-Me_2_/TiO_2_) nanocomposites were synthesized in a similar manner for comparison. The structure and properties of the nanocomposites were investigated by FTIR, UV-Vis and X-ray diffraction. In addition, the photocatalytic activities of the nanocomposite were evaluated by the degradation processes of a methylene blue (MB) solution under UV light and sunlight irradiation.

## 2. Results and Discussion

### 2.1. Fourier Transform Infrared (FTIR) Spectra

**Figure 1.**
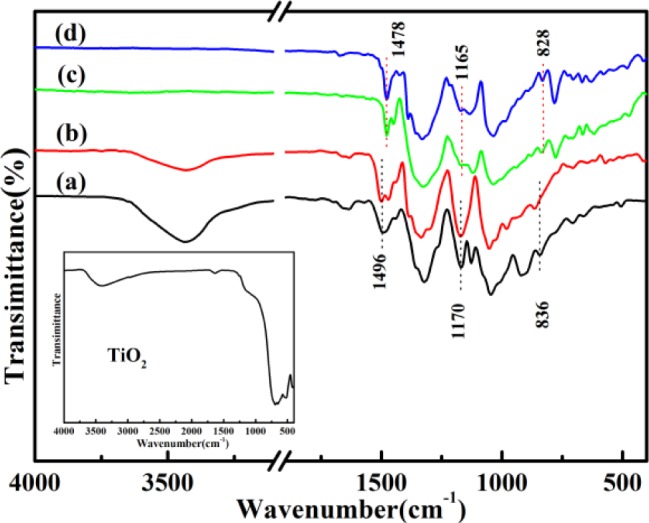
The Fourier transform infrared (FTIR), spectra of (**a**) poly(3,4-propy-lenedioxythiophene) (PProDOT)/TiO_2_; (**b**) PProDOT-Me_2_/TiO_2_; (**c**) poly(3,4-propylenedioxy-2,2′:5′,2″-terthiophene (TPT))/TiO_2_ and (**d**) poly(3,4-(2,2-dimethylenepropylenedioxy)-2,2′:5′,2″-terthiophene(TMPT))/TiO_2_. The inset shows the spectrum of nano-TiO_2_.

Figure 1 gives the FTIR spectra of solid-state synthesized PProDOT/TiO_2_, PProDOT-Me_2_/TiO_2_, poly(TPT)/TiO_2_, poly(TMPT)/TiO_2_ and nano-TiO_2_. As can be seen in Figure 1, the bands for PProDOT/TiO_2_ and PProDOT-Me_2_/TiO_2_ are as follows: ~1650, ~1490, ~1320, ~1167, ~1125, ~1045, ~920, ~838 and ~706 cm^−1^. The bands at ~1650, ~1490 and ~1320 cm^−1^ are assigned to the characteristic bands of the thiophene ring [20]. The bands appearing at ~1167, ~1125 and ~1045 cm^−1^ are associated with the C–O–C bending vibration in the propylene oxide group [21,22], while the bands at ~916, ~838 and ~706 cm^−1^ are the characteristic bands of the stretching vibrations of the C–S–C bond in the thiophene ring [23–25], which are similar to the previously reported IR spectrum of PPorDOT [26,27]. Furthermore, comparing the FTIR spectra of poly(TPT)/TiO_2_ and poly(TMPT)/TiO_2_ with that of PProDOT/TiO_2_ and PProDOT-Me_2_/TiO_2_, one can see that the positions of the main IR bands of poly(TPT)/TiO_2_ and poly(TMPT)/TiO_2_ nanocomposites are nearly identical with that of PProDOT/TiO_2_ and PProDOT-Me_2_/TiO_2_. However, the bands of the poly(TPT)/TiO_2_ and poly(TMPT)/TiO_2_ nanocomposites at ~1496, ~1170 and ~832 cm^−1^ are shifted slightly from their corresponding position to the lower wavenumber compared with that of the PProDOT/TiO_2_ and PProDOT-Me_2_/TiO_2_ nanocomposites, which indicates that the higher oxidation degree and stronger interaction between the TiO_2_ with polymer occur in the case of the poly(TPT)/TiO_2_ and poly(TMPT)/TiO_2_ nanocomposites, as compared to the PProDOT/TiO_2_ and PProDOT-Me_2_/TiO_2_ nanocomposites [28]. In addition, a close look will reveal that the characteristic peak of TiO_2_ at ~3410 cm^−1^ is appearing in the case of PProDOT/TiO_2_ and PProDOT-Me_2_/TiO_2_ nanocomposites. However, there is no characteristic peak corresponding to the TiO_2_ in the poly(TPT)/TiO_2_ and poly(TMPT)/TiO_2_ nanocomposites in Figure 1, implying that the TiO_2_ was enwrapped by the polymer [29].

## 2.2. Ultraviolet-Visible (UV-Vis) Absorption Spectra

**Figure 2.**
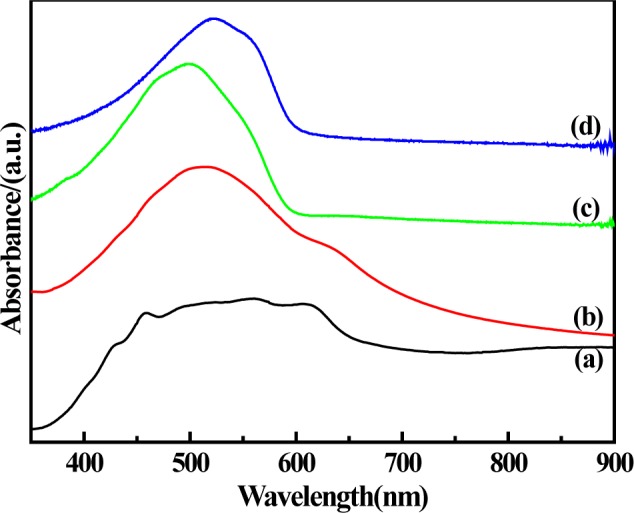
Ultraviolet-visible (UV-Vis) spectra of (**a**) PProDOT/TiO_2_; (**b**) PProDOT-Me_2_/TiO_2_; (**c**) poly(TPT)/TiO_2_ and (**d**) poly(TMPT)/TiO_2_; in *N*-methylpyrrolidone (NMP).

The UV-Vis spectra of solid-state synthesized PProDOT/TiO_2_, PProDOT-Me_2_/TiO_2_, poly(TPT)/TiO_2_ and poly(TMPT)/TiO_2_ in N-methylpyrrolidone(NMP) are shown in Figure 2. As shown in Figure 2, PProDOT/TiO_2_ displays a broad peak raging from ~430 nm to 610 nm with several shoulders at ~430, ~450, ~500, ~560 and ~610 nm, while the PProDOT-Me_2_/TiO_2_, poly(TPT)/TiO_2_ and poly(TMPT)/TiO_2_ display relatively sharp peaks raging from ~460 nm to ~610 nm with several shoulders at ~460, ~500, ~554 and ~610 nm. The absorption peaks and shoulders at ~430–560 nm are assigned to the π → π* transition of the thiophene ring [30–33], while the peaks at ~610 nm are the p-doping peaks of the polythiophene molecular chains [34]. Comparing with the absorption spectra of others, more shoulders appear in the PProDOT/TiO_2_ nanocomposite, which can be considered as the absorption peaks arising from conjugated segments having different conjugation lengths [35]. Furthermore, the peak of the PProDOT-Me_2_/TiO_2_ nanocomposite at ~500 nm is red shifted to ~523 nm in the poly(TMPT)/TiO_2_ nanocomposite. Generally, the red shift of the absorption spectrum shows the increase of the conjugated chain length [36]. Moreover, the peak of poly(TPT)/TiO_2_ appears at a relatively lower wavelength compared to the poly(TMPT)/TiO_2_, which is mostly originated from the strong interaction between the TiO_2_ particles and the poly(TMPT) [37].

### 2.3. X-ray Diffraction Patterns and *Energy Dispersive X-ray Spectroscopy* (EDX) Analysis

**Figure 3.**
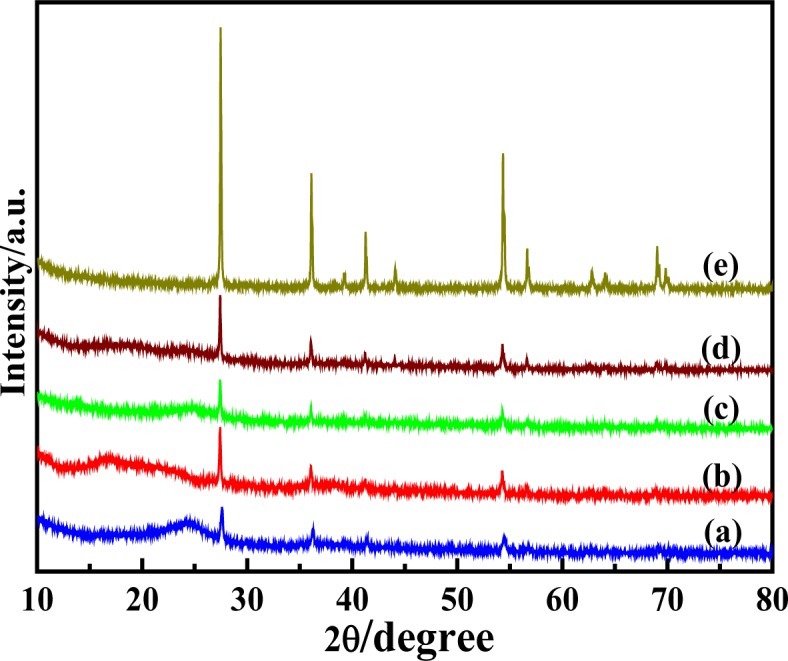
X-ray diffraction (XRD) patterns of (**a**) PProDOT/TiO_2_; (**b**) PProDOT-Me_2_/TiO_2_; (**c**) poly(TPT)/TiO_2_; (**d**) poly(TMPT)/TiO_2_; and (**e**) nano-TiO_2_.

Figure 3 shows the XRD patterns of solid-state synthesized PProDOT/TiO_2_, PProDOT-Me_2_/TiO_2_, poly(TPT)/TiO_2_, poly(TMPT)/TiO_2_ and nano-TiO_2_, respectively. As seen from Figure 3, the composites possess rather broad diffraction peaks, suggesting a small degree of crystallinity, but an amorphous structure, which is similar to other thiophene derivatives [38], and its diffraction peaks are located at about 2θ ~ 24.4° (associated with the intermolecular π → π* stacking or assigned to the (020) reflection) [39] and 2θ ~ 37.5° (assigned to the (111) reflection [40,41]). The XRD patterns of composites shows the presence of the characteristic diffraction peaks of rutile TiO_2_ (~27.6°, 36.1°, 41.3° 54.4°, 56.7° and 69.5°), suggesting the successful incorporation of rutile TiO_2_. Figure 3 implies that the poly(TMPT)/TiO_2_ nanocomposite has lower crystallinity than others. This can be associated with the introduction of dimethyl substituents in the PProDOT or poly(TPT/TiO_2_), which may decrease the crystallinity with its separation effect on the polymer main chains [42]. However, the intensity of the characteristic diffraction peaks of TiO_2_ is lower in the case of the PProDOT/TiO_2_ and poly(TPT)/TiO_2_ nanocomposites than that of PProDOT-Me_2_/TiO_2_ and poly(TMPT)/TiO_2_, suggesting that the TiO_2_ particles are uniformly embedded in the polymer matrix of PProDOT/TiO_2_ and poly(TPT)/TiO_2_ [43].

**Figure 4.**
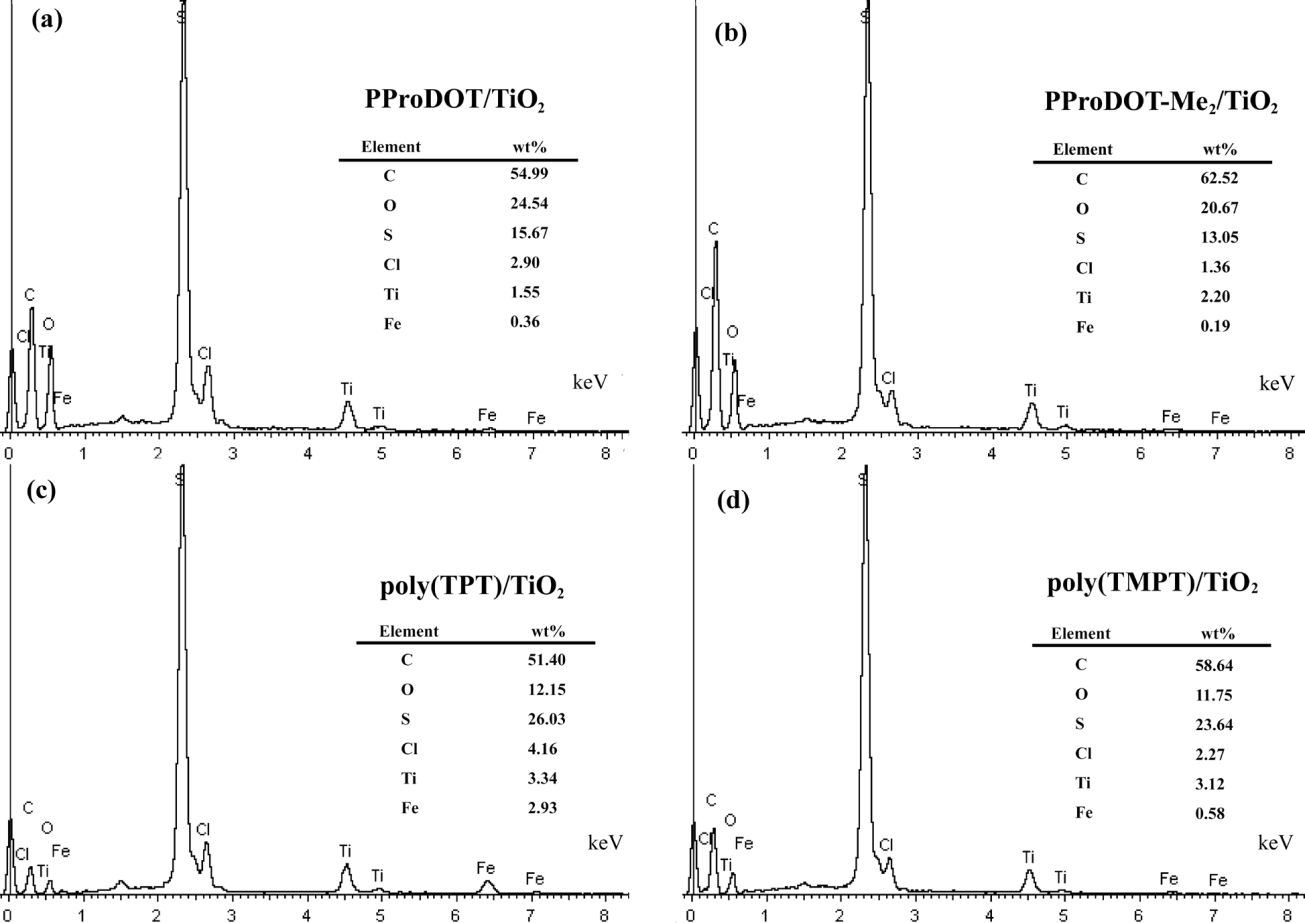
Energy dispersive X-ray spectroscopy (EDX) spectra of each composite: (**a**) PProDOT/TiO_2_; (**b**) PProDOT-Me_2_/TiO_2_; (**c**) poly(TPT)/TiO_2_; and (**d**) poly(TMPT)/TiO_2_.

The energy dispersive X-ray spectroscopy (EDX) analysis on each composite is presented in Figure 4. As shown in Figure 4, the elements of C, O, S, Cl, Ti and Fe are observed. Additionally, the quantitative analysis of the result of EDX indicates that the wt% of Ti in each nanocomposite is 1.55%, 2.20%, 3.34% and 3.12%, respectively, which implies that poly(TPT)/TiO_2_ and poly(TMPT)/TiO_2_ have a higher wt% of TiO_2_ than that of PProDOT/TiO_2_ and PProDOT-Me_2_/TiO_2_.

### 2.4. Morphology

**Figure 5.**
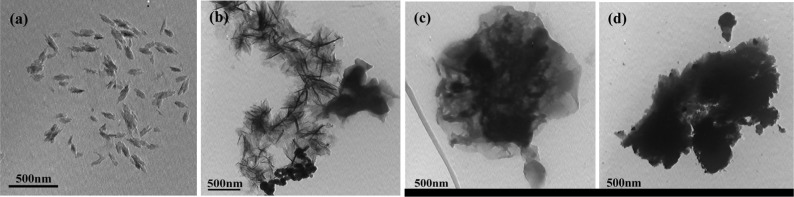
Transmission electron microscopy (TEM) images of (**a**) PProDOT/TiO_2_; (**b**) PProDOT-Me_2_/TiO_2_; (**c**) poly(TPT)/TiO_2_; and (**d**) poly(TMPT)/TiO_2_.

Figure 5 represents the transmission electron microscopy (TEM) images of solid-state synthesized PProDOT/TiO_2_, PProDOT-Me_2_/TiO_2_, poly(TPT)/TiO_2_ and poly(TMPT)/TiO_2_. As shown in Figure 5, all the nanocomposites display a sponge-like morphology, except from the PProDOT/TiO_2_ nanocomposite. Additionally, the TiO_2_ particles (dark-shaded nanoparticles) are found to be entrapped in polymer (light shaded) matrix. These results reveal that the TiO_2_ particles are not simply mixed up or blended with the polymer, suggesting that the TiO_2_ particles are embedded in the polymer matrix, which is quite in accordance with the results of the XRD analysis.

### 2.5. Photocatalytic Properties

**Figure 6.**
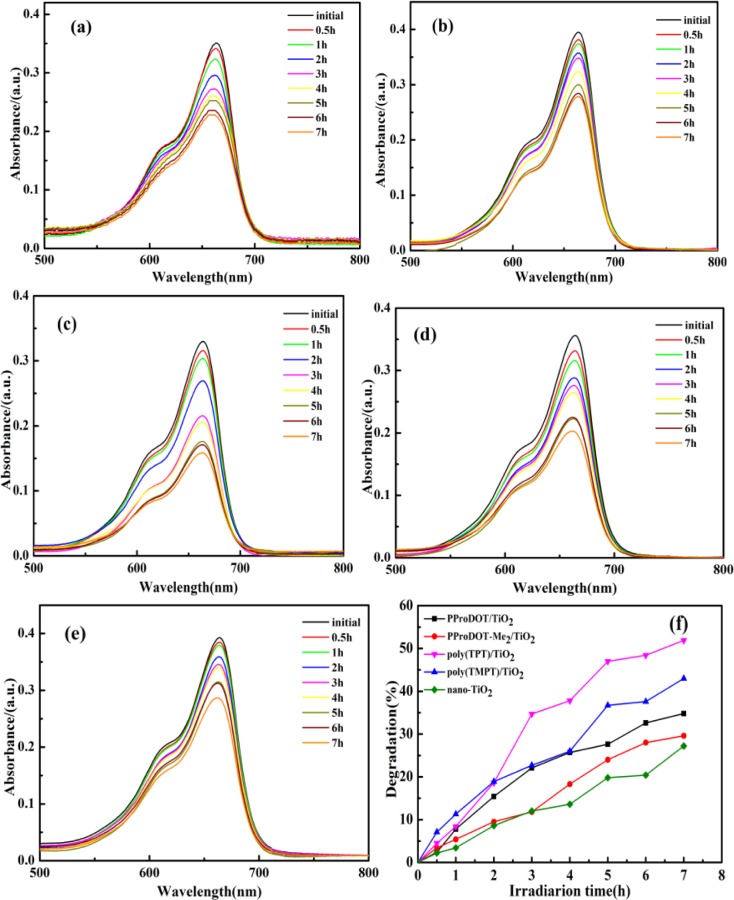
The UV-Vis absorption spectra of methylene blue (MB) dyes by photocatalysis for different irradiation times under UV light irradiation: (**a**) PProDOT/TiO_2_; (**b**) PProDOT-Me_2_/TiO_2_; (**c**) poly(TPT)/TiO_2_; (**d**) poly(TMPT)/TiO_2_; (**e**) Nano-TiO_2_; and (**f**) the degradation efficiency of the MB dyes (catalyst concentration: 0.4 mg/mL; initial concentration of dyes: 1 × 10^−5^ M).

**Figure 7.**
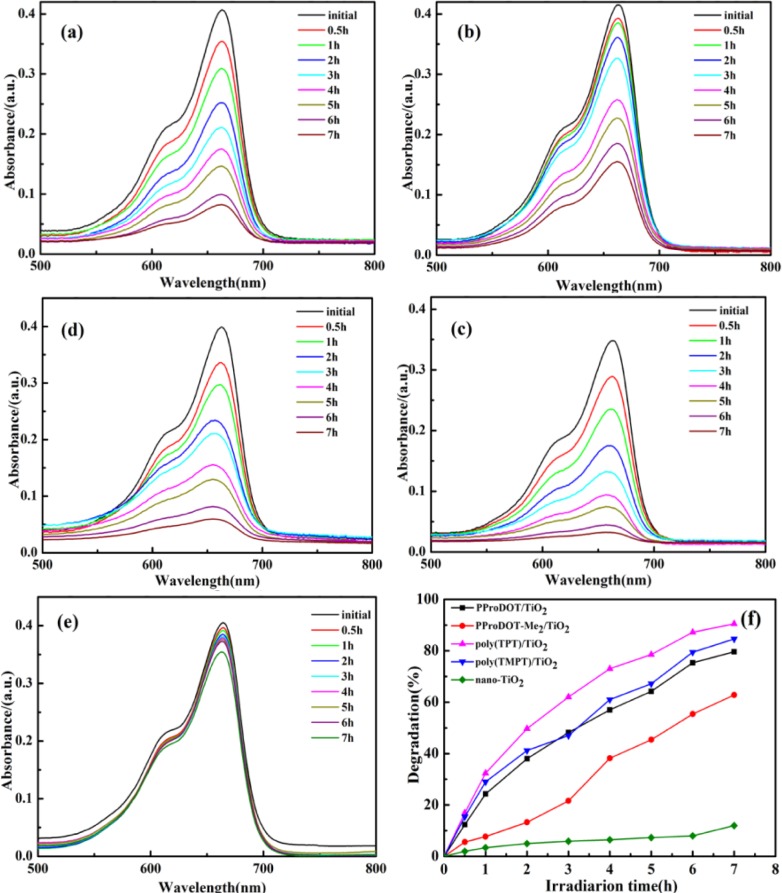
The UV-Vis absorption spectra of MB dyes by photocatalysis for different irradiation times under sunlight irradiation: (**a**) PProDOT/TiO_2_; (**b**) PProDOT-Me_2_/TiO_2_; (**c**) poly(TPT)/TiO_2_; (**d**) poly(TMPT)/TiO_2_; (**e**) Nano-TiO_2_; and (**f**) the degradation efficiency of the MB dyes (catalyst concentration: 0.4 mg/mL; initial concentration of dyes: 1 × 10^−5^ M).

Figure 6 shows the photocatalytic degradation of methylene blue (MB) dye with the presence of the PProDOT/TiO_2_, PProDOT-Me_2_/TiO_2_, poly(TPT)/TiO_2_ and poly(TMPT)/TiO_2_ nanocomposites and pure nano-TiO_2_ as catalysts under UV light at different irradiations time, while Figure 7 indicates the photocatalytic degradation of MB dye in the presence of the above composites and the pure nano-TiO_2_ as the catalyst under natural sunlight at different irradiations time. Generally, the adsorption/desorption equilibrium is an important preliminary step in the photocatalytic degradation process. Therefore, prior to irradiation, the MB solution was magnetically stirred in the dark for 30 min to ensure the establishment of the adsorption/desorption equilibrium. As can be seen in Figures 6 and 7, the diminished characteristic band of MB dye at ~660 nm after 7 h under UV light and natural sunlight indicates that the MB has been degraded by all the nanocomposites and pure nano-TiO_2_. Additionally, the degradation efficiency of each nanocomposite is 32.5%, 29.6%, 51.5% and 44.4% under UV light for PProDOT/TiO_2_, PProDOT-Me_2_/TiO_2_, poly(TPT)/TiO_2_ and poly(TMPT)/TiO_2_, respectively, while the degradation efficiency of each nanocomposite is 79.6%, 62.8%, 90.5% and 84.6% under natural sunlight for PProDOT/TiO_2_, PProDOT-Me_2_/TiO_2_, poly(TPT)/TiO_2_ and poly(TMPT)/TiO_2_, respectively. In addition, the degradation efficiency of nano-TiO_2_ under UV light and natural sunlight is 27% and 11.6%, respectively. It is easy to see that the degradation efficiency of the MB in the presence of poly(TPT)/TiO_2_ and poly(TMPT)/TiO_2_ is higher than that of PProDOT/TiO_2_ and PProDOT-Me_2_/TiO_2_. Meanwhile, the degradation efficiency increased up to two and three times compared to PProDOT/TiO_2_ and PProDOT-Me_2_/TiO_2_, respectively, under the same conditions, which suggests that the poly(TPT)/TiO_2_ and poly(TMPT)/TiO_2_ nanocomposites are more effective photocatalysts for the degradation of MB than the PProDOT/TiO_2_ and PProDOT-Me_2_/TiO_2_ nanocomposites.

Based on previous reports, the photocatalytic degradation of MB under UV or visible light irradiation is mainly related to the generation of reactive hydroxy and hydroperoxy radicals [44–46]. Herrmann *et al.* investigated the TiO_2_/UV photocatalytic degradation of MB in aqueous heterogeneous suspensions, and a detailed degradation pathway was determined by a careful identification of intermediate products, in particular aromatics, whose successive hydroxylations led to the aromatic ring opening. Furthermore, they found that TiO_2_/UV-based photocatalysis was simultaneously able to oxidize the MB with an almost complete mineralization of carbon and of nitrogen and sulfur heteroatoms into CO_2_, NH_4_^+^, NO_3_^−^ and SO_4_^2−^, respectively [47]. According to the reports, the hydroxy and hydroperoxy radicals produced by the action of photocatalyst are capable of oxidizing MB molecules at the surface layer of photocatalyst. MB molecules tend to be adsorbed by the surface of the catalyst. This process can be enhanced by controlling the surface charge of the photocatalyst. Moreover, MB is a cationic dye, and a negatively charged photocatalyst surface accelerates the adsorption of the MB and, consequently, the photo degradation process [44,45].

As can be seen in Figure 7, almost complete degradation of the MB has been achieved 7 h under natural light irradiation using nanocomposites as the photocatalyst, except from the PProDOT-Me_2_/TiO_2_ nanocomposites. In addition, the degradation efficiency of both poly(TPT)/TiO_2_ and poly(TMPT)/TiO_2_ are higher than that of PProDOT/TiO_2_ and PProDOT-Me_2_/TiO_2_. To summarize, the poly(TPT)/TiO_2_ and poly(TMPT)/TiO_2_ nanocomposites had higher degradation efficiencies than the PProDOT/TiO_2_ and PProDOT-Me_2_/TiO_2_ nanocomposites under both light sources. Moreover, the degradation efficiency of all nanocomposites under sunlight are much higher than under UV light, which indicates that the nanocomposites are more effective photocatalysts for the degradation of MB under the higher intensity of sunlight irradiation than UV irradiation.

**Figure 8.**
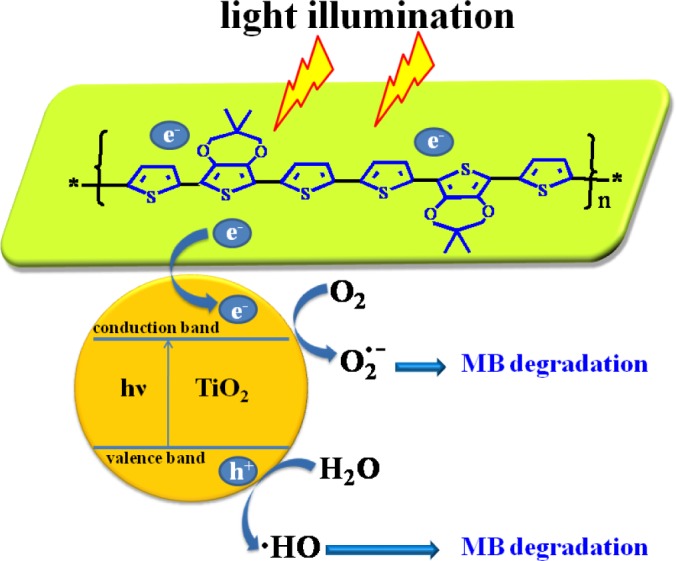
A schematic illustration of the photocatalytic activity of a nanocomposite (take the poly(TMPT)/TiO_2_ nanocomposite for example).

Figure 8 shows the schematic mechanism of MB dye degradation to explain the photocatalytic activity of the nanocomposite catalyst under sunlight. According to the previous report, TiO_2_ particles can absorb UV light to create electrons (e^−^) in the conduction band and holes (h^+^) in the valence band [48], respectively. If the electrons and holes cannot be captured in time, they will recombine with each other within a few nanoseconds, which will reduce the photocatalytic efficiency of TiO_2_. However, in the case of composites, due to the existence of the interface between polymer and TiO_2_, separated electrons and holes have little possibility to recombine again. This ensures higher charge separation efficiency and better photo-oxidation capacity for the nanocomposite. In addition, the polymer can absorb the visible light and produces an electron (e^−^) that transfers to the conduction band of TiO_2_ [48,49]. The amount of ^•^OH and O_2_^•−^ formed in the case of composites is more than that with TiO_2_ alone as a photocatalyst. Moreover, MB molecules can transfer from solution to the catalyst’s surface and be adsorbed with an offset face-to-face orientation via π-π conjugation between MB and aromatic regions of the polymer, and therefore, the adsorptivity of MB on polymer increases compared to that of MB on bare TiO_2_, which makes the composites have a higher efficiency in the photodegradation of MB compared to TiO_2_ alone as a photocatalyst.

## 3. Experimental Section

### 3.1. Materials

2-(tributylstannyl)-thiophene, *N*-bromo-succinimide (NBS), 3,4-propylenedioxythiophene (ProDOT), 3,4-(2,2-dimethylenepropylenedioxy)thiophene(ProDOT-Me_2_) and anhydrous iron(III) chloride were obtained from Aldrich (Tokyo, Japan) and used as received. Pd (PPh_3_)_4_ was synthesized according to the literature [50,51]. The nano-TiO_2_ (rutile, lipophilic, with an average size of 100 nm, Shanghai Aladdin Reagent Company, Shanghai, China) and all other chemicals were used as received without further purification.

### 3.2. Preparation of Monomers

**Scheme 1:**
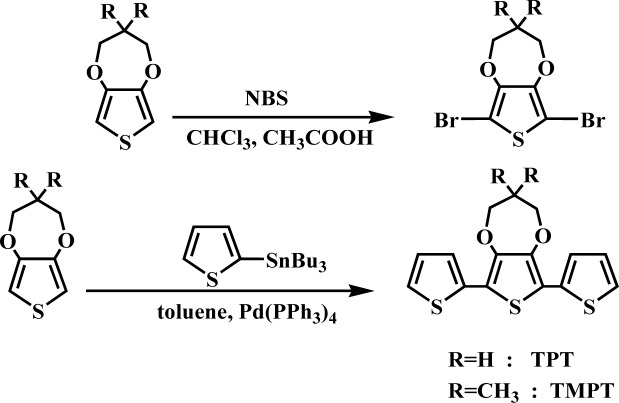
Synthesis of monomers (TPT and TMPT).

3,4-propylenedioxy-2,2′:5′,2″-terthiophene(TPT) and 3,4-(2,2-dimethylenepropylene-dioxy)-2,2′:5′,2″-terthiophene(TMPT) were synthesized according to the procedure given in [42], as shown in Scheme 1.

### 3.3. Preparation of Composites

A typical solid-state synthesis procedure of the composite was as follows: a mixture of 0.3 g (0.9 mmol) 3,4-propylenedioxy-2,2′:5′,2″-terthiophene (TPT) and 15 mg TiO_2_ in 3 mL chloroform were ultrasonicated for 30 min to facilitate the monomer in adsorbing onto the surface of TiO_2_. After ultrasonication, the mixture was allowed to evaporate the chloroform, then the mixture was put in a mortar, and 0.61 g (3.6 mmol) anhydrous iron(III) chloride (FeCl_3_) were added to this mixture, then ground for 1 h. Then, the mixture was washed with chloroform, ethanol and distilled water, respectively, until the filtrate was colorless, finally drying the powder under vacuum at 60 °C for 48 h. The obtained composite was denoted as poly(TPT)/TiO_2_. Poly(TMPT)/TiO_2_ was synthesized in a similar manner by keeping the ratio of [TMPT]/[FeCl_3_] at 1:4.

The other composites, poly(3,4-propylenedioxythiophene)/TiO_2_ nanocomposite(PProDOT/TiO_2_) and poly(3,4-(2,2-dimethylenepropylenedioxy)thiophene)/TiO_2_ composite(PProDOT/TiO_2_), were obtained in a similar manner by keeping the ratio of [monomer]/[FeCl_3_] at 1:4.

### 3.4. Structure Characterization

The Fourier transform infrared (FTIR) spectra of the composite were obtained by using a BRUKERQEUINOX-55 Fourier transform infrared spectrometer (Billerica, MA, USA) (frequency range: 4000–500 cm^−1^). The UV-Vis spectra and photocatalytic activity of the samples were recorded on a UV-Visible spectrophotometer (UV4802, Unico, Franksville, WI, USA). X-ray powder diffraction (XRD) patterns were obtained by using a Bruker AXS D8 diffractometer (Bruker AXS Co., Karlsruhe, Germany), with a monochromatic Cu-Ka radiation source (λ = 0.15418). The scan range (2θ) was 5°–80°. Transmission electron microscopy (TEM) experiments were carried out in a Hitachi 2600 electron microscope (Tokyo, Japan). The samples for TEM measurements were prepared by placing a few drops of sample ethanol suspension on copper supports.

### 3.5. Measurement of Photocatalytic Activities

The photocatalytic activities of nanocomposites were performed using MB as degraded materials in quartz tubes under UV light and natural sunlight irradiation. FSL MW1-Y15 (Royal Dutch Philips Electronics Ltd., Amsterdam, The Netherlands) was used as the irradiation source (λ = 254 nm) located in a light infiltrated chamber. Before irradiation, the suspension was stirred magnetically for 30 min in dark conditions until an adsorption-desorption equilibrium was established. Then, the suspensions were irradiated by light sources. Under natural sunlight investigations, all experiments were done inside a laboratory in an open atmosphere in the month of September. The photodegradation efficiency (*R*, %) was calculated by use of the following equation: *R* = [*C*_0_ − *C*/*C*_0_] (where *C*_0_ represents the concentration of the dye before illumination and *C* denotes the concentration of dye after a certain irradiation time, respectively).

## 4. Conclusions

In this paper, the polyterthiophene derivatives/TiO_2_ nanocomposites, such as poly(3,4-propylenedioxy-2,2′:5′,2″-terthiophene)/TiO_2_ nanocomposite (poly(TPT)/TiO_2_) and poly(3,4-(2,2-dimethylenepropylenedioxy)-2,2′:5′,2″-terthiophene)/TiO_2_ nanocomposite (poly(TMPT)/TiO_2_), were synthesized by a simple solid-state method. The poly(3,4-propylenedioxythiophene)/TiO_2_ nanocomposite (PProDOT/TiO_2_) and poly(3,4-2,2-dimethylenepropylenedioxythiophene)/TiO_2_ nanocomposite (PProDOT-Me_2_/TiO_2_) were synthesized by the same method for comparison. The results showed that the higher oxidation degree and the strong interaction between TiO_2_ and polymer occurred in the case of poly(TPT)/TiO_2_ and poly(TMPT)/TiO_2_ compared with that of PProDOT/TiO_2_ and PProDOT-Me_2_/TiO_2_. This phenomenon mainly resulted from the linear growth tendency of terthiophene-type monomers, which made the whole polyterthiophene-type chain grow regularly and led to an enhancement of the electronic and optical properties of the nanocomposites. The results also indicated that TiO_2_ had no effect on the crystallinity of the polymer, but TiO_2_ can be embedded in the polymer matrix, which implied that the solid-state method can be an effective method for preparing such composite materials. Furthermore, the UV-Vis photodegradation efficiency of MB by these composites showed that the poly(TPT)/TiO_2_ and poly(TMPT)/TiO_2_ nanocomposites had higher degradation efficiency than that of the PProDOT/TiO_2_ and PProDOT-Me_2_/TiO_2_ nanocomposites, which resulted from the positive effect of the higher oxidation degree of the polyterthiophene derivatives/TiO_2_ nanocomposites, as well as the strong interaction between TiO_2_ and the polyterthiophene derivatives than that of PProDOT/TiO_2_ and PProDOT-Me_2_/TiO_2_. The photodegradation studies suggested that the polyterthiophene derivatives/TiO_2_ nanocomposites were more effective photocatalysts for the degradation of MB under UV and sunlight irradiation. All nanocomposites were more effective photocatalysts as compared to bare TiO_2_, because the polymers acted as light-harvesting species, and the polymers displayed a positive role in charge separation, except the adsorption effect of polymers for MB via π-π conjugation between MB and aromatic regions of the polymer.
